# Detection of altered pain facilitatory and inhibitory mechanisms in patients with knee osteoarthritis by using a simple bedside tool kit (QuantiPain)

**DOI:** 10.1097/PR9.0000000000000998

**Published:** 2022-04-01

**Authors:** Masashi Izumi, Yoshihiro Hayashi, Ryota Saito, Shota Oda, Kristian Kjær Petersen, Lars Arendt-Nielsen, Masahiko Ikeuchi

**Affiliations:** a Department of Orthopaedic Surgery, Kochi Medical School, Kochi University, Nankoku, Kochi, Japan; b Department of Rehabilitation, Kochi Medical School Hospital, Kochi University, Nankoku, Kochi, Japan; c Center for Innovative and Translational Medicine, Kochi Medical School, Kochi University, Nankoku, Kochi, Japan; d Department of Health Science and Technology, Center for Neuroplasticity and Pain (CNAP), Department of Health Science and Technology, School of Medicine, Aalborg University, Aalborg, Denmark; e Department of Medical Gastroenterology, Mech-Sense, Aalborg University Hospital, Aalborg, Denmark

**Keywords:** Pain, Quantitative sensory testing, Pressure pain threshold, Temporal summation of pain, Conditioned pain modulation, Osteoarthritis

## Abstract

**Purpose:**

Altered pain facilitatory and inhibitory mechanisms have been recognized as an important manifestation in patients with chronic pain, and quantitative sensory testing (QST) can act as a proxy for this process. We have recently developed a simple bedside QST tool kit (*QuantiPain*) for more clinical use. The purpose of this study was to investigate its test–retest reliability and to evaluate its validity compared with the laboratory-based QST protocols in patients with knee osteoarthritis (OA).

**Methods:**

*QuantiPain* consists of 3 items: “pressure algometer” (for pressure pain thresholds [PPTs]), “pinprick” (for temporal summation of pain [TSP]), and “conditioning clamp” (for conditioned pain modulation [CPM]). In experiment-A, intrarater and interrater test–retest reliabilities were investigated in 21 young healthy subjects by using interclass correlation coefficient (ICC). In experiment-B, 40 unilateral painful patients with OA and 40 age-matched, healthy control subjects were included to compare the bedside tool kit against the computerized pressure algometry.

**Results:**

In experiment-A, excellent to moderate intrarater and interrater reliabilities were achieved in PPT and TSP (ICC: 0.60–0.92) while the agreements of CPM were good to poor (ICC: 0.37–0.80). In experiment-B, localized and widespread decrease of PPT, facilitated TSP, and impaired CPM was found by using the bedside tool kit in patients with OA compared with controls (*P* < 0.05). The data were significantly correlated with the established laboratory-based tools (*R* = 0.281–0.848, *P* < 0.05).

**Conclusion:**

*QuantiPain* demonstrated acceptable test–retest reliability and assessment validity with the sensitivity to separate patients with painful OA from controls, which has a potential to create more practical approach for quantifying altered pain mechanisms in clinical settings.

## Abstract 2

Supplemental Digital Content is Available in the Text.

A simple bedside quantitative sensory testing tool kit demonstrated acceptable reliability and assessment validity for detecting altered pain facilitatory and inhibitory mechanisms in patients with painful osteoarthritis.

## 1. Introduction

Pain perception is always subjective and thereby challenging to objectively quantify; however, several biomarkers related to mechanisms, neural activity, and susceptibility offer the possibility of this quantification.^40^ Quantitative sensory testing (QST) is a psychophysical method and can act as a proxy to investigate the functional states of the somatosensory system by evaluating thresholds or responses to standardized stimuli.^34^ Quantitative sensory testing has been received much attention with an indirect measure of “pain sensitization” or “pain de-sensitization” in the peripheral and central pain pathways.^8,21^ Quantitative sensory testing modalities are categorized as static or dynamic procedures that enables to monitor sensory functions regarding different features of transmission and control of pain.^6^

Pressure pain threshold (PPT) has been a widely used static QST for assessing painful musculoskeletal pain disorders.^10^ Localized or regional hyperalgesia may indicate sensory dysfunction at the peripheral level, whereas widespread hyperalgesia to remote nonpainful area seems to be a proxy for abnormalities in central nervous system processing.^10^ Dynamic QST includes temporal summation of pain (TSP) as a phenomenon in which repeated nociceptive stimuli cause an increase in pain perception^24^ and conditioned pain modulation (CPM) assessing the endogenous pain modulatory pathways by the “pain inhibits pain” phenomenon.^49^

Altered pain facilitatory and inhibitory mechanisms assessed by QST have been recognized as an important manifestation in patients with chronic pain.^3^ In addition, QST has been used to predict chronic postoperative pain^15,28,47^ and response to analgesic effects,^30,48^ and a recent review demonstrated that the most frequent reported predictors were PPTs, TSP, and CPM.^32^ Among multiple chronic pain conditions, osteoarthritis (OA) is one of the most intensively studied diseases, and accumulating evidence suggests that localized and widespread decrease of PPT, facilitated TSP, and impaired CPM are key contributors to chronic pain in subgroups of patients with OA.^1,4,22,23,39^

Nevertheless, laboratory-based QST tools were complicated, expensive, training necessary, and time consuming,^17,33^ and hence, it has not become a popular pain assessment for routine clinical applications. To solve this problem, we have recently developed a simple bedside tool kit (*QuantiPain*) for evaluating PPT, TSP, and CPM. This study mainly evaluated its (1) test–retest reliability in healthy subjects and (2) validity compared with the laboratory-based QST protocols in patients with knee OA. In addition, we investigated the relationship between the bedside QST data and chronic pain–associated questionnaires that has been receiving attention for contributing pain mechanisms in patients with OA.^41,51^

## 2. Materials and methods

This study comprised 2 experiments. First, test–retest reliability of *QuantiPain* assessment was investigated in young healthy subjects (experiment-A). Second, validity of *QuantiPain* assessment was evaluated in healthy subjects and patients with knee OA compared with laboratory-based QST tools (experiment-B).

The study protocol was approved by the Institutional Review Board of Kochi Medical School (No. 31–61). All participants received verbal explanation of this study and provided written informed consent before the investigation. This study was conducted in compliance with the tenets of the Declaration of Helsinki.

### 2.1. Participants

In experiment-A, 21 healthy, pain-free subjects (10 females, age 27 years [20–38]) were participated. In experiment-B, 40 patients with knee OA (32 females, age 67 years [49–84 years]) suffering for at least 3 months from unilateral knee pain while walking, and age-matched 40 healthy control subjects (17 female, age 61 years [27–84 years]) were recruited. Patients with bilateral knee OA were included only if 1 knee was pain free. In both experiments, subjects and patients who were diagnosed as having painful musculoskeletal disorders (except knee OA), neurological disorders, psychiatric diseases, skin disorders at examination sites, and taking pain killers 24 hours before the experiment were excluded.

### 2.2. Experiment-A

**Figure 1.**
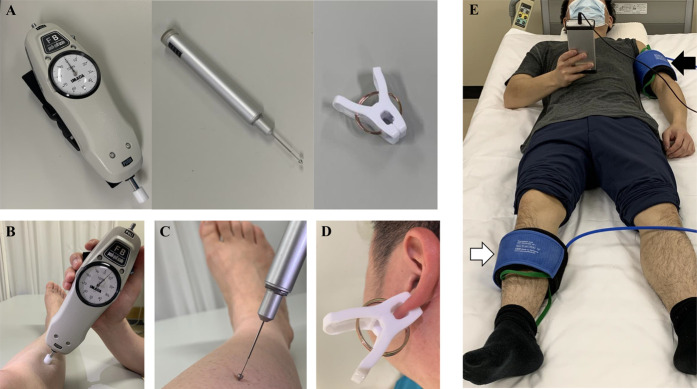
(A) Overview of the 3 items included in the bedside quantitative sensory testing tool kit, (B) pressure algometer, (C) pinprick, (D) conditioning clamp, and (E) cuff pressure algometry. White arrow: cuff for test stimulus. Black arrow: cuff for conditioning stimulus. The participants continuously rate their pain using a Visual Analogue Scale controller.

*QuantiPain* consists of 3 items: “pressure algometer” (for pressure pain thresholds, PPTs), “pinprick” (for TSP), and “conditioning clamp” (for CPM) (Fig. 1A). The pressure algometer is made by just mounting a 1-cm^2^ plastic probe on the tip of a hand-held analogue mechanical force gauge (available for measurement from 0 to 100 N, IMADA Co, LTD, Aichi, Japan). The custom pinprick (Takei Scientific Instrument Co, LTD, Niigata, Japan) incorporates a 60 g mobile weight that provides identical repeated painful stimuli through the conical tip (3 mm in maximal diameter). The conditioning clamp applies extrasegmental tonic pain stimulus (4.5 kg force applied to approximately 175 mm^2^). Durability of the conditioning clamp was preliminarily confirmed that the force did not decrease after pinching 200 times.

The participants were carefully familiarized with the methods and then laid in a supine position on the bed and took 5 minutes rest before the start of assessment. Pressure pain threshold was measured by using pressure algometer on tibialis anterior muscle (5 cm distal to the tibial tuberosity) and deltoid muscle (5 cm distal to the acromion) without monitoring of pressure acceleration itself, but it was increased roughly at a speed of 5 N/s, and subjects were instructed to verbally report the point at which pressure changed into pain (Fig. 1B). The force gauge has a peak mode which enables to keep the PPT value accurately. TSP was evaluated by 10 consecutive stimuli (1 second interval between the stimulus) by using pinprick on the same sites of PPT measurement and dorsum of hand (midpoint of the third and fourth metacarpal bone) (Fig. 1C). The TSP effect was calculated as the difference in the pain Visual Analogue Scale (VAS) (mm) between the first and the last stimuli, as previously reported.^18,31^ The VAS was anchored with “no pain” and “worst pain imaginable” at 0 mm and 100 mm, respectively. Conditioned pain modulation was evaluated by using conditioning clamp for applying tonic pain stimulus by pinching the earlobe for 60 seconds^19^ (Fig. 1D). When pain VAS of the earlobe became more than 60 mm, PPT was evaluated on contralateral tibialis anterior and deltoid muscle. The CPM effect was calculated as the percent change [(conditioning/baseline × 100) − 100] and difference [conditioning—baseline] of the PPT, as previously recommended.^46^ The QST was performed unilaterally, and the test side was randomized in each healthy subject. Each measure except CPM was recorded 3 times, and the average measurement was used for analysis. Regarding each CPM measurement, conditioning pain was applied once and PPTs were recorded twice during the single conditioning period, and the average of the PPT was used for the assessment. All tests were performed at a similar time in the evening.

Two trained experimenters (Y.H. and R.S.) performed QST with an interval of a week. Intrarater and interrater test–retest reliabilities of the PPT, TS, and CPM at each measurement site were analyzed by using interclass correlation coefficient (ICC).

### 2.3. Experiment-B

Validity of *QuantiPain* assessment was evaluated compared with laboratory-based QST tools on the same day. In patients with OA, bilateral assessments were performed for PPT and TSP, but CPM was evaluated only on the affected side. In healthy controls, the assessment side was randomized in each subject. The sequence of QST sessions by using *QuantiPain* or laboratory tools was also randomized. Before QST, the patients with OA were interviewed about the duration of their knee pain and average of the pain VAS (mm) at rest and while walking in the past week. They were also asked to complete chronic pain–associated questionnaires including Central Sensitization Inventory (CSI),^20^ Hospital Anxiety and Depression Scale (HADS),^50^ and Pain Catastrophizing Scale (PCS).^38^

*QuantiPain* was used identically with the experiment-A for PPT (medial joint space of the knee and tibialis anterior muscle), TSP (tibialis anterior muscle), and CPM (tibialis anterior muscle) assessments.

As an established laboratory-based tool, a digital hand-held algometer (SBMEDIC, Hörby, Sweden) mounted with a 1-cm^2^ probe was used for PPT recordings.^4,45^ Pressure was increased gradually at a rate of 30 kPa until the pain threshold was reached and the participants pressed a stop button.

Temporal summation of pain and CPM were measured by using cuff algometry (Cortex, NociTech and Aalborg University, Denmark), which is another all-in-one laboratory-based tool for QST (Fig. 1E).^13,29,37^ This consists of a 13-cm-wide tourniquet cuff, a computer-controlled air compressor, and an electronic VAS (Aalborg University, Aalborg, Denmark). The cuff was connected to the compressor and wrapped around the lower leg and was automatically inflated at a rate of 1 kPa/s. The pressure-induced pain intensity was recorded with the electronic 100-mm VAS and sampled at 10 Hz. The subjects were instructed to rate the VAS pain intensity continuously and to press a hand-held pressure release button when the pain was intolerable. This pressure value was defined as pressure tolerance threshold (PTT).

For TSP measurement, the cuff pressure stimuli were applied 10 times with a 1 second interstimulus interval and duration. The applied pressure was equal to the PTT.^37^ The participants were instructed to continuously rate their pain on a VAS. The TSP effect was calculated as the difference in the VAS between the first and the tenth stimuli.

For CPM measurement, another cuff applied to the contralateral upper arm was promptly inflated to a pressure corresponding to 70% of the PTT as conditioning stimuli.^37^ The cuff on the ipsilateral lower leg was then inflated at a rate of 1 kPa/s. The participants were instructed to rate the pain on their lower leg. Similar to the *QuantiPain* session, the CPM effect was calculated as the percent change and difference of pressure detection threshold (a pressure value corresponding to the VAS = 10 mm) with and without the conditioning stimuli. Pain VAS for the conditioning tonic pain (*QuantiPain*: earlobe and cuff algometry: upper arm) was also evaluated.

### 2.4. Statistical analysis

Sample size was determined according to some previous studies. Increasing evidence suggest that a large effect size when comparing QST parameters in patients with OA with healthy subjects^5^ and at least 24 subjects were needed in each group to detect a significant difference based on a large effect size (Cohen d = 0.8) and a significant level at 0.05 and a power of 80%.^4,15^ This was similar to our previously published reliability study on QST profiles.^11^ Most of the data did not pass Shapiro–Wilk test for normal distribution and were presented in the median and interquartile range. In experiment-A, intrarater and interrater data of the PPT, TSP, and CPM were compared by using the Wilcoxon signed-rank test. Intrarater reliability was assessed by using ICC (1, k) for data from test 1 and test 2 by a single experimenter (R.S.). Interrater reliability was also assessed by using ICC (3, k) for data from the 2 experimenters. Values less than 0.5, between 0.5 and 0.75, between 0.75 and 0.9, and greater than 0.90 are indicative of poor, moderate, good, and excellent reliability, respectively.^16^ In addition, standard error of measurement and smallest real difference were calculated as absolute measure for the reliability of the QST parameters^44^ (see supplementary table 1, available at http://links.lww.com/PR9/A155).

In experiment-B, PPTs were analyzed per each assessment site. The Kruskal–Wallis test was performed on PPT and TSP using the factors of group (control, OA-affected side, and OA-contralateral side). The Mann–Whitney *U* test with the Bonferroni correction was used for post hoc comparisons when the Kruskal–Wallis test showed significant factors.

Comparison of CPM between groups was analyzed by the Mann–Whitney *U* test. The differences of QST tools were analyzed by the Wilcoxon signed-rank test in each group. Furthermore, a ranked distribution analysis of the CPM effects^2^ was added for comparison between the groups and tools, and frequency of antinociceptive (increase PPT) or pronociceptive (decrease PPT) reaction against the conditioning stimulus was evaluated by using the χ^2^ test.

Correlations of QST data between *QuantiPain* and the laboratory-based tools were analyzed in patients with OA and control subjects by using the Spearmen correlation coefficient. Moreover, correlations between *QuantiPain* data, knee pain VAS, and chronic pain–associated questionnaires were investigated in patients with OA. All analyses were performed with SPSS version 26.0 software (IBM Corp. Armonk, NY), and *P* < 0.05 indicated statistical significance.

## 3. Results

### 3.1. Experiment-A

The median (interquartile range) results of the reliability analyses and ICCs are presented in Table 1 for intrarater agreement and in Table 2 for interrater agreement, respectively. The conditioning pain VAS for the CPM assessment was similar between the 2 experimenters (median: 70.0 mm), which seemed relevant to a recent methodology for evoking mechanically induced CPM.^11^ Regarding intrarater assessment, no significant differences of QST data between tests 1 and 2 were seen and excellent to moderate reliability was achieved except CPM which was evaluated by the difference of PPT on deltoid muscle. By contrast, interrater assessment showed significant difference of PPT on both sites and TSP on deltoid muscle, although reliability of these tests was excellent. Agreements of CPM were moderate to poor when it was evaluated by the percent change or difference of PPT on both sites.

**Table 1:** Results of intrarater reliability analysis.

	Test 1	Test 2	Wilcoxon (*P*)	ICC (1, k) [95% CI]
PPT (N)				
Tibialis anterior	51.3 [37.2–60.3]	47.0 [38.0–53.8]	0.677	0.90 [0.76–0.96]
Deltoid	30.5 [24.0–36.7]	33.0 [20.8–37.9]	0.955	0.94 [0.84–0.97]
TSP (mm)				
Tibialis anterior	16.0 [8.0–24.5]	11.0 [6.0–20.0]	0.196	0.84 [0.61–0.93]
Deltoid	15.0 [6.5–27.0]	11.0 [6.0–28.0]	0.265	0.77 [0.43–0.90]
Hand	15.0 [7.5–24.5]	12.0 [6.5–21.5]	0.575	0.60 [0.13–0.83]
CPM				
Tibialis anterior				
Percent change (%)	17.8 [9.8–26.0]	21.8 [9.6–28.5]	0.677	0.76 [0.41–0.90]
Difference (N)	6.7 [4.5–12.8]	9.0 [5.2–13.7]	0.794	0.80 [0.52–0.92]
Deltoid				
Percent change (%)	32.5 [15.5–50.0]	20.3 [10.7–43.9]	0.313	0.67 [0.20–0.87]
Difference (N)	8.0 [4.3–16.3]	6.0 [4.5–9.7]	0.266	0.39 [-0.47–0.75]

Data from tests 1 and 2 are presented as median [interquartile range]. ICC (1, k) was presented with 95% CI.

CI, confidence interval; ICC, interclass correlation coefficient; PPT, pressure pain threshold; TSP, temporal summation of pain; CPM, conditioned pain modulation.

**Table 2:** Results of interrater reliability analysis.

	Experimenter 1	Experimenter 2	Wilcoxon (*P*)	ICC (3, k) [95% CI]
PPT (N)				
Tibialis anterior	48.4 [39.4–56.3]	55.7 [49.8–71.5]	**0.0002**	0.92 [0.80–0.97]
Deltoid	31.8 [22.2–37.6]	39.7 [25.5–46.8]	**0.0002**	0.90 [0.76–0.96]
TSP (mm)				
Tibialis anterior	13.5 [8.0–22.0]	9.0 [3.0–20.0]	0.357	0.86 [0.64–0.94]
Deltoid	14.0 [5.3–21.3]	8.0 [2.0–22.5]	**0.038**	0.91 [0.78–0.96]
Hand	14.0 [7.5–24.3]	10.0 [5.0–21.0]	0.578	0.71 [0.28–0.88]
CPM				
Tibialis anterior				
Percent change (%)	17.0 [9.2–30.5]	10.4 [8.3–19.1]	0.23	0.61 [0.04–0.84]
Difference (N)	6.8 [4.4–12.5]	7.0 [4.4–13.0]	0.972	0.37 [-0.56–0.74]
Deltoid				
Percent change (%)	27.9 [15.1–38.9]	25.9 [16.1–43.8]	0.876	0.72 [0.31–0.89]
Difference (N)	7.8 [4.8–12.5]	10.0 [5.2–14.6]	0.179	0.45 [-0.36–0.78]

Data from experimenters 1 and 2 are presented as median [interquartile range]. ICC (3, k) was presented with 95% CI.

Bold indicates significant difference between the experimenters.

CI, confidence interval; ICC, interclass correlation coefficient; PPT, pressure pain threshold; TSP, temporal summation of pain; CPM, conditioned pain modulation.

### 3.2. Experiment-B

Demographic data of the patients with knee OA and healthy control subjects are presnted in Table 3. There was no significant difference of age between groups, but the OA group included more women than the control group. Four patients in the OA group showed radiological bilateral knee OA; however, they had no pain in the contralateral knee.

**Table 3:** Demographic data of patients with knee osteoarthritis and control subjects in experiment-B.

Variable	Patients with knee OA	Control subjects
N	40	40
Age (y)	69 [58–74]	66 [48–73]
Sex, n (%)		
Male	8 (20)	23 (57)
Female	32 (80)	17 (43)
Pain duration, mo	36.0 [4.5–168.0]	0
VAS at rest, mm	0.0 [0.0–19.3]	0
VAS on walking, mm	48.0 [31.8–67.3]	0
CSI	20.5 [11.3–27.8]	—
HADS		
Anxiety	3.5 [2.0–6.3]	—
Depression	4.0 [3.0–9.0]	—
PCS	16.0 [8.0–25.0]	—

Data are presented as median [interquartile range].

CSI, Central Sensitization Inventory; HADS, Hospital Anxiety and Depression Scale; OA, osteoarthritis; PCS, Pain Catastrophizing Scale; VAS; Visual Analogue Scale.

#### 3.2.1. Quantitative sensory testing measures by different technologies

**Figure 2.**
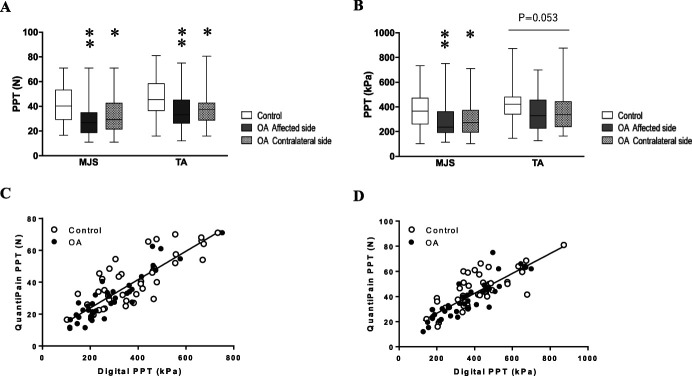
Results of pressure pain threshold (PPT). (A) *QuantiPain* (pressure algometer), (B) laboratory tool (digital algometer), (C) correlation between using both tools measured on MJS, and (D) correlation between using both tools measured on TA. *P < 0.05 vs control, **P < 0.01 vs control. MJS; medial joint space, TA; tibialis anterior muscle.

PPTs recorded on medial joint space of the knee and tibialis anterior muscle were bilaterally lower in patients with OA compared with control subjects. This finding was comparable between using *QuantiPain* (Fig. 2A) and the digital algometer (Fig. 2B), but the difference did not reach statistical significance when using the digital algometer on tibialis anterior muscle. Moreover, excellent correlations were observed between PPTs evaluated by *QuantiPain* and the digital algometer on medial joint space of the knee (affected side: *R* = 0.848, *P* < 0.0001, Fig. 2C. and contralateral side: *R* = 0.819, *P* < 0.0001) and tibialis anterior muscle (affected side: *R* = 0.815, *P* < 0.0001, Fig. 2D. and contralateral side: *R* = 0.807, *P* < 0.0001).

**Figure 3.**
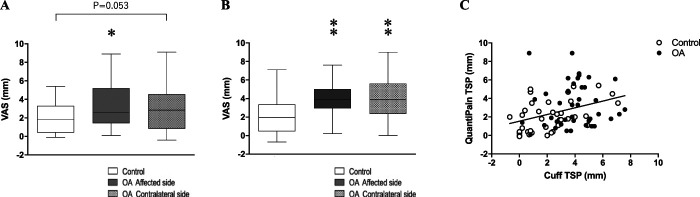
Results of temporal summation of pain (TSP). (A) *QuantiPain* (pinprick), (B) laboratory tool (cuff algometry), and (C) correlation between using both tools. *P < 0.05 vs control, **P < 0.01 vs control.

Enhancement of TSP was observed in both groups; however, it was significantly facilitated in patients with OA compared with control subjects when evaluated by *QuantiPain* (Fig. 3A) and cuff algometry (Fig. 3B). A moderate correlation was seen between TSP evaluated by both systems (affected side: *R* = 0.447, *P* < 0.0001, Fig. 3C. and contralateral side: *R* = 0.284, *P* = 0.0107).

**Figure 4.**
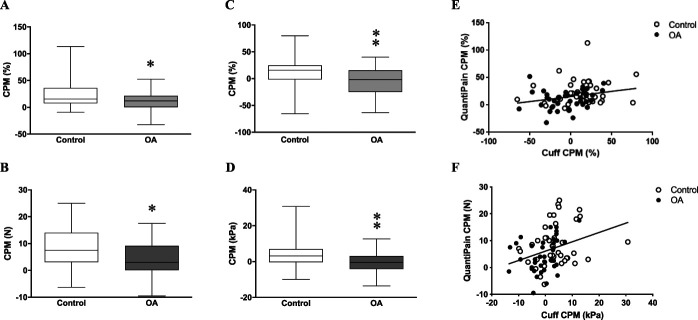
Results of conditioned pain modulation (CPM). (A) *QuantiPain* (pressure algometer with conditioning clamp) analyzed by percent change of PPT, (B) *QuantiPain* analyzed by difference of PPT, (C) laboratory tool (cuff algometry) analyzed by percent change of PPT, (D) laboratory tool analyzed by difference of PPT, (E) correlation between using both tools analyzed by percent change of PPT, and (F) correlation between using both tools analyzed by difference of PPT. *P < 0.05 vs control, **P < 0.01 vs control. PPT, pressure pain threshold.

Conditioned pain modulation was significantly impaired in patients with OA compared with control subjects when evaluated by *QuantiPain* (Figs. 4A and B) and cuff algometry (Figs. 4C and D). A weak but significant correlation was seen between the CPM evaluated by both systems (percent change of PPT: R = 0.281, *P* = 0.0116, Fig. 4E and difference of PPT: R = 0.3786, *P* = 0.0005, Fig. 4F). The percent change of PPT was significantly greater by using *QuantiPain* compared with cuff algometry in patients with OA (Wilcoxon; *P* = 0.001), but not in control subjects (Wilcoxon; *P* = 0.077). Pain VAS for the conditioning tonic pain was significantly greater in *QuantiPain* (earlobe) than cuff algometry (upper arm) in both groups (patients with OA: 80.0[70.0–80.0] mm vs 64.1[48.0–70.0] mm; Wilcoxon; *P* < 0.000001 and control subjects: 67.0[60.0–80.0] mm vs 62.0[48.3–70.0] mm; Wilcoxon; *P* = 0.00028). The conditioning pain VAS was higher in patients with OA than control subjects in *QuantiPain* session (Mann–Whitney, *P* = 0.005), but this difference was not seen when using cuff algometry (Mann–Whitney, *P* = 0.972).

**Figure 5.**
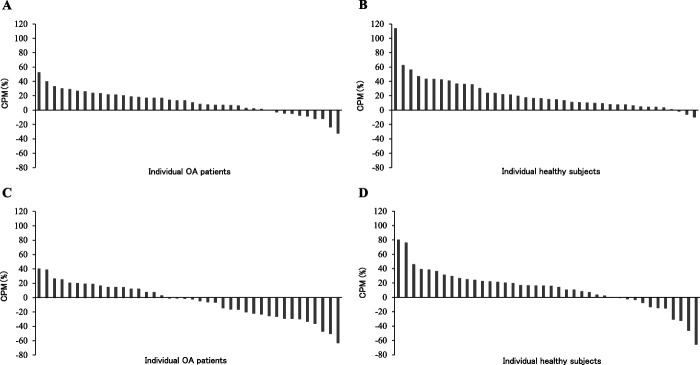
Ranked distribution analysis of the CPM effect (presented as percent change of PPT). Positive and negative values show antinociceptive and pronociceptive reaction against the conditioning tonic pain stimulus, respectively. (A) *QuantiPain* (patients with OA), (B) *QuantiPain* (healthy subjects), (C) cuff algometry (patients with OA), and (D) cuff algometry (healthy subjects). CPM, conditioned pain modulation; PPT, pressure pain threshold; OA, osteoarthritis.

The ranked distribution analysis demonstrated that 75% of the patients with OA showed antinociceptive reaction while it was 93% for the control subjects assessed by *QuantiPain* (χ^2^; *P* = 0.034, Figs. 5A and B). By contrast, cuff algometry categorized 43% of the patients with OA and 70% of the control subjects as antinociceptive, respectively (χ^2^; *P* = 0.013, Figs. 5C and D).

#### 3.2.2. Quantitative sensory testing and chronic pain–associated questionnaires

Correlations between *QuantiPain* data, knee pain VAS, and chronic pain–associated questionnaires were displayed in Supplementary table 2 (available at http://links.lww.com/PR9/A155). Ipsilateral PPTs on medial joint space and tibialis anterior muscle were negatively correlated with pain VAS at rest (*R* = −0.35, −0.33), on walking (*R* = −0.38, −0.44), and PCS (*R* = −0.37, −0.37). Ipsilateral TSP was associated with CSI (*R* = 0.37), whereas CPM was not correlated with all questionnaires. PCS was correlated with CSI (*R* = 0.53) and HADS anxiety (*R* = 0.32). CSI was also correlated with HADS anxiety (*R* = 0.60). Among QST parameters, PPT on tibialis anterior muscle was correlated with TSP (*R* = −0.38) and CPM (*R* = 0.48), but no significant correlation was observed between TSP and CPM.

## 4. Discussion

This study demonstrated that *QuantiPain* had acceptable test–retest reliability. In addition, this tool kit successfully detected localized and widespread decrease of PPT, facilitated TSP, and impaired CPM in patients with OA compared with controls, and the data were significantly correlated with laboratory-based QST assessment. Because the tool kit has promising benefits such as being cheap, easy to use, and portable, it seems to have enough potential for clinical application.

### 4.1. Test–retest reliability of the bedside quantitative sensory testing tool kit

Regarding PPT and TSP, good to excellent intrarater and interrater reliabilities were confirmed except TSP assessed on dorsum of hand being moderate, which were comparable with established pressure algometry and cuff algometry reported in the previous studies.^12,42^ A plausible reason of lower reliability of TSP was the hand being thinner and had more mobile skin with less deep tissues compared with other assessment sites. However, rapid assessment on hand seems to be a great advantage for clinical application.

As recognized, CPM is one of the most unstable QST test partly because of its methodological complexity and variability of test and conditioning stimulus, and hence, intrarater and interrater reliabilities were lower in general.^26,36^ *QuantiPain* showed, however, moderate to good intrarater reliability of CPM effect except the difference of PPT on deltoid, and the ICC (1, k) was comparable with a similar bedside tool reported as 0.67 to 0.72.^19^ As for the interrater assessment, moderate reliability was confirmed when evaluated the CPM effect with percent change of PPT, whereas the reliability became poor when evaluated with the difference of PPT. However, the reliability of CPM assessment by *QuantiPain* does not seem to be far less than cuff algometry of which ICC (3, k) was documented as 0.47 to 0.73 in a previous study.^11^

### 4.2. Quantitative sensory testing measures by different technologies

Localized and widespread hyperalgesia, facilitated TSP, and impaired CPM were found in patients with OA, which were similar results from laboratory-based tools in this study, and consistent with current understanding of altered pain mechanisms in patients with OA.^8,21^

Looking at each parameter, PPT measured by our pressure algometer showed excellent correlation with the use of the digital algometer. Although it is not surprising as PPT measurement itself was technically almost identical, this finding suggests that roughly increasing pressure at a speed of 5 N/s and verbally indicated thresholds were acceptable measurement for bedside testing.

For TSP, the pinprick stimulation was a different procedure compared with cuff algometry, ie, pinprick mainly stimulated localized superficial tissues while cuff algometry compressed larger area of deep tissues. According to a previous experimental study, TSP was likely to be facilitated by painful stimuli to deep structures rather than superficial tissues.^24^ Nevertheless, there was a significant moderate correlation between TSP measured by both systems. One possible explanation of this accordance results from structural characteristics of our pinprick device that incorporates a 60 g mobile weight in the body and conical tip. Compared with filaments used in other studies,^17,34^ this device may provide more effective stimuli to superficial and deep structures for facilitating TSP. Because the assessment is extremely easy, this procedure will become a good option for bedside TSP testing.

Impaired CPM in patients with OA was indicated by *QuantiPain* and cuff algometry; however, the correlation of CPM data between both systems was weaker compared with that of PPT and TSP. As mentioned, CPM is a highly variable assessment in general, so the main reason of the discordance was probably derived from the difference of test and conditioning stimulus between the 2 systems. In addition, pain intensity of conditioning stimulus was significantly higher when assessed by *QuantiPain* than cuff algometry in both patients with OA and healthy controls. Because recent studies supported that more painful conditioning stimulus evokes more CPM effect,^11,25,27^ management of the cramping force to earlobe for individual subject might be needed to achieve a more stable result.

The ranked distribution analysis of the CPM provided further interesting information in this study. Frequency of antinociceptive and pronociceptive reaction was significantly different not only between the groups but also between the assessment devises. Because the CPM effects are usually biphasic (ie, antinociceptive and pronociceptive) especially in patients with chronic pain having sensitization, simple analysis using representative values (e.g., average) of the cohorts may be at a risk of overlooking true outcomes. Moreover, it is important that both healthy subjects and patients with OA could individually demonstrate a “scattered,” not a “binary” CPM response. In this regard, our new approach would help understanding the characteristics of CPM assessment.

### 4.3. Quantitative sensory testing and chronic pain–associated questionnaires

Relationship between QST data and chronic pain–associated questionnaires still remains unclear, but assumed to be weak in recent reports. Walton et al.^43^ showed that depression, catastrophizing, and kinesiophobia were able to explain small variance in local PPT in people with mechanical neck pain. Coronado et al.^7^ showed that CSI was associated with resilience, anxiety, and negative effects but not with PPT on remote sites in patients with shoulder pain. In patients with knee OA, Gervais-Hupe et al.^9^ reported that CSI was weakly correlated with decreased PPT locally and remotely, and with CPM, but not with TSP. They also mentioned that the CSI is more strongly associated with psychological factors than QST results. Consistent with these reports, this study showed weak correlations between some, but limited QST data and questionnaires (local PPT vs PCS and TSP vs CSI); however, the impact was much smaller than that of PCS vs CSI or CSI vs HADS anxiety. It makes sense because psychophysical tests and psychological questionnaires are not identical measures, although part of patients with chronic pain has overlapped abnormalities detected by both items.

No significant correlation was observed between TSP and CPM. Temporal summation of pain is often facilitated, and CPM is often impaired in patients with chronic pain compared with healthy subjects.^3^ However, a recent human experimental study revealed that these 2 dynamic QST assessments were not associated,^14^ which was similar to the findings in this study.

### 4.4. Comparison with other bedside quantitative sensory testing tool kit

Development of easy-to-use bedside QST tool kit has been a hot topic for phenotyping patients with chronic pain. Koulouris et al.^17^ developed a bedside equipment for evaluating patients with neuropathic pain. They confirmed moderate test–retest reliability of the assessment, and the results were highly correlated with laboratory-based QST variables. Reimer et al.^33^ presented another bedside equipment and demonstrated that sensory loss, thermal hyperalgesia, and mechanical hyperalgesia were nominated as bedside cluster assessment. Their new equipment will help stratification of patients with neuropathic pain; however, it seems a bit complicated and concerned that most of the parameters are assessed by static QST with suprathreshold stimulus response.

By contrast, our concept for development of *QuantiPain* is “copying laboratory-based mechanistic QST as simple as possible at bedside,” and hence, the algometer was used for analyzing subthreshold stimulus response and detecting PPT. Moreover, TSP and CPM, known as the 2 measure paradigms of dynamic QST, are incorporated by adding quite simple items (pinprick and conditioning clamp) to evaluate overreaction and dysfunction of the central nervous system. This concept would be partly supported by a latest report developing a bedside tool kit for assessing sensitization in patients with chronic OA knee pain.^35^ They included mechanical pinprick sensitivity, dynamic mechanical allodynia, pressure pain sensitivity, TSP, and CPM for analysis and detected 46% of patients showed signs of sensitization.

### 4.5. Limitations

There were some limitations to be noted when interpreting the results of this study. First, the number of patients with OA and healthy subjects was relatively small and recruited from single institution because this is a preliminary study investigating test–retest reliability and validity of *QuantiPain*. Second, 80% females were included in patients with OA while only 43% females in control group in experiment 2, which might affect the outcome of comparison between the 2 groups. However, the main purpose of this comparison was to confirm the validity of QuantiPain compared with laboratory-based QST tools; therefore, the effects of sex difference probably worked equally when using both tools. In addition, correlations of QST variables between both tools were analyzed by using the data from patients with OA and controls together for minimizing the effects of sex difference. Third, *QuantiPain* focused on mechanistic approach of QST and mainly targeted on deep somatic pain. Lack of thermal and light touch stimulus sensitivity might be a disadvantage of *QuantiPain,* especially for neuropathic pain evaluation; however, we prioritized simple protocols than comprehensive sensory testing in this study.

## 5. Conclusion

The presented, simple bedside tool kit demonstrated acceptable test–retest reliability and assessment validity that would be capable of evaluating painful patients. Although this has not become a complete alternative of laboratory-based tools and further research is warranted for improving reliability, the tool kit has a potential to create more practical approach for quantifying altered pain mechanisms in clinical settings.

## Disclosures

M. Izumi received grants from Shionogi & Co., Ltd. during the conduct of this study. The remaining authors have no conflicts of interest to declare.

This study was financially supported by Shionogi & Co, Ltd (Osaka, Japan).

## Appendix A. Supplemental digital content

Supplemental digital content associated with this article can be found online at http://links.lww.com/PR9/A155.

## Supplementary Material

SUPPLEMENTARY MATERIAL
