# Supplementary material for: Detection of altered pain facilitatory and inhibitory mechanisms in patients with knee osteoarthritis by using a simple bedside tool kit (QuantiPain)
Source: Pain Rep. 2022 Apr 1;7(3):e998. doi: 10.1097/PR9.0000000000000998 (PMC8984378; doi:10.1097/PR9.0000000000000998)
Supplement: SUPPLEMENTARY MATERIAL [file painreports-7-e998-s001.pdf]

Supplementary table 1. Standard error of measurement (SEM) and smallest real difference (SRD) of the QST parameters. Data from inter-rater reliability analysis were used. Calculations: SEM= intra-individual standard deviation*√(1-ICC), SRD = 1.96 * SEM * √2. The SRD indicates the changes in value that cannot be interpreted as random scatters between two measurements in an individual, but has to be assigned to change due to a treatment or intervention effect.

	**SEM**	**SRD**
**PPT (N)**		
Tibialis anterior	4.4	12.2
Deltoid	3.7	10.1
**TSP (mm)**		
Tibialis anterior	5.2	14.5
Deltoid	4.0	11.0
Hand	7.3	20.1
**CPM**		
Tibialis anterior		
Percent change (%)	9.6	26.7
Difference (N)	4.7	13.0
Deltoid		
Percent change (%)	11.2	31.1
Difference (N)	3.5	9.6

Supplementary table 2. Correlations between QuantiPain data, knee pain VAS, and chronic-pain-associated questionnaires. Upper column; Spearman's correlation coefficient, Lower column; P-value. '-'means no significant correlation. Abbreviations: PPT; pressure pain threshold, MJS; medial joint space, TA; tibialis anterior muscle, TSP; temporal summation of pain, CPM; conditioned pain modulation (presented as difference of PPT), VAS; visual analogue scale, CSI; Central Sensitization Inventory, HADS-A; Anxiety score of Hospital Anxiety and Depression Scale, HADS-D; Depression score of Hospital Anxiety and Depression Scale, PCS; Pain Catastrophizing Scale.

	PPT MJS	PPT TA	TSP	CPM	VAS rest	VAS walk	CSI	HADS-A	HADS-D	PCS
**PPT MJS**		0.82	-	0.39	-0.35	-0.38	-	-	-	-0.37
		<0.0001		0.013	0.026	0.017				0.022
**PPT TA**	0.82		-0.38	0.48	-0.33	-0.44	-	-	-	-0.37
	<0.0001		0.017	0.002	0.037	0.005				0.022
**TSP**	-	-0.38		-	-	-	0.37	-	-	-
		0.017					0.022			
**CPM**	0.39	0.48	-		-	-	-	-	-	-
	0.013	0.002								
**VAS rest**	-0.35	-0.33	-	-		0.54	-	-	-	-
	0.026	0.037				0.0003				
**VAS walk**	-0.38	-0.44	-	-	0.54		-	-	-	-
	0.017	0.005			0.0003					
**CSI**	-	-	0.37	-	-	-		0.6	-	0.53
			0.022					<0.0001		0.0006
**HADS-A**	-	-	-	-	-	-	0.6		0.33	0.32
							<0.0001		0.041	0.049
**HADS-D**	-	-	-	-	-	-	-	0.33		-
								0.041		
**PCS**	-0.37	-0.37	-	-	-	-	0.53	0.32	-	
	0.022	0.022					0.0006	0.049		
